# Identification of Long Noncoding RNAs lnc-DC in Plasma as a New Biomarker for Primary Sjögren's Syndrome

**DOI:** 10.1155/2020/9236234

**Published:** 2020-10-15

**Authors:** Yanhong Chen, Yongqiang Chen, Beibei Zu, Jia Liu, Li Sun, Chen Ding, Duping Wang, Xing Cheng, DeLiang Yang, Guoping Niu

**Affiliations:** Department of Clinical Laboratory, XuZhou Central Hospital, China

## Abstract

**Objective:**

To evaluate the plasma levels of lnc-DC in primary Sjögren's syndrome (pSS) patients and investigate the potential associations between lnc-DC and disease activity.

**Methods:**

In this study, we recruited 358 enrollments, including 127 pSS patients without immune thrombocytopenia (ITP), 22 pSS patients with ITP, 50 systemic lupus erythematosus (SLE) patients, and 50 patients with rheumatoid arthritis (RA) and 109 healthy individuals, from Xuzhou Central Hospital. The expression of anti-SSA and anti-SSB was detected by enzyme-linked immunosorbent assay (ELISA). Spearman rank correlation test was used to analyze the relationship between lnc-DC and pSS activity. pSS activity was measured by anti-SSA, anti-SSB antibody, erythrocyte sedimentation rate (ESR), and *β*_2_-microglobulin levels. The receiver operating characteristic (ROC) curve was used to determine the diagnostic performance of plasma lnc-DC for pSS.

**Results:**

Compared with healthy controls, SLE and RA patients, the lnc-DC expression levels were significantly elevated in pSS patients (*P* < 0.001), especially in pSS patients with ITP (*P* < 0.001). As expected, we also found that the lnc-DC expression positively correlated with anti-SSA (*R*^2^ = 0.290, *P* < 0.001), anti-SSB (*R*^2^ = 0.172, *P* < 0.001), ESR level (*R*^2^ = 0.076, *P* = 0.002), and *β*_2_-microglobulin level (*R*^2^ = 0.070, *P* = 0.003) in pSS patients. ROC curves showed that plasma lnc-DC in pSS patients had an AUC 0.80 with a sensitivity of 0.75 and specificity of 0.85 at the optimum cutoff 1.06 in discriminating SLE and RA patients. In addition, the combination of lnc-DC and anti-SSA/SSB (AUC: 0.84, sensitivity: 0.79, specificity: 0.90) improved significantly the diagnostic ability of pSS patients from SLE and RA patients. In the efficacy monitoring study, levels of plasma lnc-DC were dramatically decreased after treatment (*P* < 0.001).

**Conclusion:**

These findings highlight that plasma lnc-DC as a novel biomarker for the diagnosis of pSS and can be used to evaluate the therapeutic efficacy of pSS underwent interventional therapy.

## 1. Introduction

Primary Sjögren's syndrome (pSS), more often seen in women, is a multifactor and organ-specific autoimmune disease [1, 2]. The main clinical symptoms of pSS are eye and oral dryness, following with other manifestations such as skin dryness and immune thrombocytopenia (ITP) [3, 4]. Like other autoimmune diseases, both environmental and genetic factors contribute to the onset of pSS [5, 6]. Accumulating evidence has demonstrated that multiple molecules including and development of pSS [7, 8]. ITP is an immune-mediated disease that characterized by impaired platelet production or platelet more destruction, resulting in platelet decreased and varying degree of bleeding risk [9]. ITP is commonly associated with autoimmune diseases such as systemic lupus erythematosus (SLE) [10, 11]. However, the pathogenesis and molecular diagnosis of pSS with or without ITP are yet to be elucidated. Our study explores the clinical and immunological characteristics of ITP in patients with pSS, suggesting that pSS patients with ITP expressed higher level of lnc-DC compared to pSS patients without ITP. And we aim to explore plasma biomarkers in pSS patients which could contribute to better diagnosis and prognosis.

Characterized as a subtype of noncoding RNAs with more than 200 nucleotides in length [12, 13], long noncoding RNAs (lncRNAs) are widely involved in various physiological and pathological processes mostly by functioning at transcriptional or posttranscriptional control [14, 15], including cancers [16], immune diseases [17], cardiovascular diseases [18–20], and cardio-metabolic diseases [21]. Piling evidence showed that lncRNAs exist stably in human body fluids including urine and plasma, thereby acting as sensitive prognostic and diagnostic biomarkers in cancers and cardiovascular diseases [22–24]. lnc-DC, identified by Wang et al., is a specific lncRNA that exclusively expressed in dendritic cells (DCs) which mediates the differentiation of DCs and the activation of T cells [25]. However, it remains uncertain whether lnc-DC in plasma can effectively diagnose pSS. Thus, in this study, we evaluate the plasma levels of lnc-DC in pSS patients and investigate their potential value for pSS diagnosis.

## 2. Materials and Methods

### 2.1. Study Subjects

A total of 127 primary Sjögren's syndrome (pSS) patients, 22 pSS patients with immune thrombocytopenia (ITP), and 109 healthy controls were collected in the physical examination central of XuZhou Central Hospital, June 1, 2018, to December 1, 2019. All pSS patients were according to the 2002 US-EU Consensus Group criteria [26], and all ITP patients fulfilled the diagnosis criteria according to the American Society of Hematology guidelines [9]. Healthy controls have no history of autoimmune diseases and have never been treated with immunosuppressive drugs. The sampling method was biopsy sampling. In order to analyze the specificity of lnc-DC in patients, 100 patients from our hospital were selected as a control group, including 50 systemic lupus erythematosus (SLE) patients and 50 rheumatoid arthritis (RA) patients, and their lnc-DC expression levels were analyzed. This study has been approved by relevant agencies and is feasible for implementation.

### 2.2. Extraction of Plasma

Use ethylenediaminetetraacetic acid anticoagulation tube as the collection device to collect 7 ~ 10 mL of peripheral blood of patients. Blood samples were centrifuged at 1500 g for 10 min, and 12000 g, 4°C for 10 min. The plasma samples were divided into several parts and then placed at -80°C for storage.

### 2.3. Enzyme-Linked Immunosorbent Assay (ELISA)

Anti-SSA and anti-SSB in the plasma were quantified using the ELISA kits (Sterlitech co, Beijing, China) according to the manufacturer's protocol. The absorbance of the samples at 405 nm is using a microplate reader (Biotek USA).

### 2.4. Quantitative Reverse Transcription Polymerase Chain (qRT-PCR) Reaction

Total RNA was extracted using PrimeScript™ RT regent kit (Invitrogen), while scientific cDNA reverse transcription was performed, followed by qRT-PCR according to the biological system protocol. The primers of lnc-DC are as follows: R-CCCTAAGATCGTCCCTTCC, F-CAACCCCTCTTCCCTGCC. The reactions involved were 96-optical plates treated at 95°C for 5 minutes, followed by 42 cycles at 95°C for 10 seconds, 30 seconds at 60°C, and 72°C for 20 seconds. lnc-DC is calculated using the relative expression of the endogenous2^-*ΔΔ*ct^ control method of standardization.

### 2.5. Data Collection

The clinical data involved in this study include specific laboratory test results, patient medical history information, specific treatment methods, and other medical records. The clinical performance of the patient is analyzed, including symptoms related to skin bleeding and mucous petechiae in pSS patients. When conducting an immunological characteristic test, the detection analysis indicators involved include the following several parts: C-reactive protein (CRP), erythrocyte sedimentation rate (ESR), complement 3 (C3), and complement 4 (C4), at the same time, the patients with analysis of antibody and immune globulin, disease activity in patients with pSS evaluation theoretical basis for the European anti rheumatoid glen syndrome disease activity index (ESSDAI) evaluation way [27]. Patients with hemorrhagic manifestations of the severity of bleeding are described by the ITP-specific assessment tools (ITP-bat) [28].

### 2.6. Statistical Analysis

Analysis of variance (ANOVA) was used for statistical analysis of data. This method can be determined whether there is a significant difference between the healthy controls and pSS patients. The pre- and posttest comparison was carried out using Bonferroni's test. The correlation between lnc-DC and clinical features was analysis by Spearman rank correlation coefficient test. Receiver operating characteristic curve (ROC) and area under curve (AUC) were used as the assessment indicators of sensitivity and specificity of pSS biomarkers for lnc-DC analysis. The data processing software used was the GraphPad Prism 7 software (GraphPad software, Inc., San Diego, CA). Mean ± standard deviation was used to describe the distribution of normal distribution quantitative data. Independent sample *t*-test was used to compare the differences between the two groups. One-way ANOVA was used to compare the differences between multiple groups. Median (quartile) was used to describe the distribution of nonnormal distribution quantitative data, Mann–Whitney *U* test was used to compare the differences between groups; frequency and composition ratio were used to describe the distribution of qualitative data, and chi-squared test or Fisher exact probability method was used to compare the differences between groups. All the tests were bilateral, and the difference was statistically significant (*P* < 0.05).

## 3. Results

### 3.1. The Level of lnc-DC Increased in pSS Patients as Compared with Healthy Controls and Other Autoimmune Diseases

**Figure 1:**
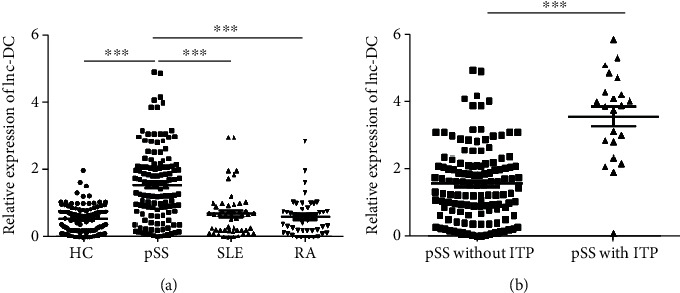
The relative expression of lnc-DC in HCs and pSS with or without ITP, SLE, and RA patients that was determined by qRT-PCR. ^∗^*P* < 0.05; ^∗∗∗^*P* < 0.001, by one-way ANOVA with Bonferroni's test and 2-tailed unpaired *t*-test.

The expressions of lnc-DC in 109 healthy controls (HC), 50 SLE, 50 RA, and 127 pSS patients were analyzed by qRT-PCR. As shown in Figure 1(a), the levels of lnc-DC were significantly elevated in pSS patients than those in HC, SLE, and RA patients (*P* < 0.001). Moreover, the levels of plasma lnc-DC in pSS patients with immune thrombocytopenia (ITP) increased dramatically than pSS patients without ITP (*P* < 0.001). A comparison of basic characteristics, the clinical manifestations, and laboratory findings of pSS patients with and without ITP is shown in Table 1.

### 3.2. The Plasma lnc-DC Level Is Positively Correlative with pSS Clinical Characteristics

**Figure 2:**
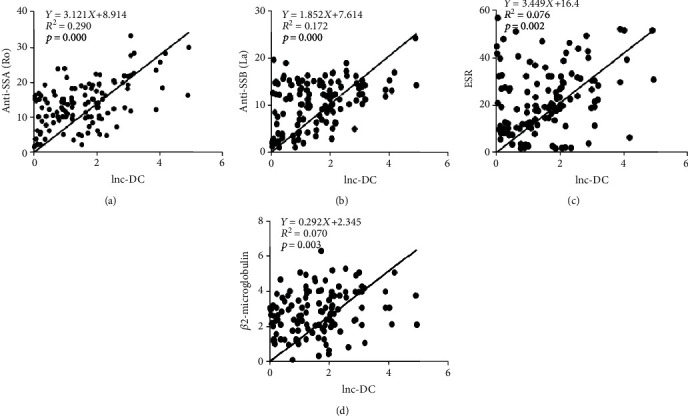
lnc-DC and the correlativity of pSS patients with certain clinical characteristics, lnc-DC, and 127 patients with pSS in the correlation between some clinical characteristics, including anti-SSA (a), anti-SSB (b), the ESR (c), and *β*_2_-microglobulin (d). The correlation analysis used the Spearman rank correlation coefficient test.

To explore the relationship between the lnc-DC expression and the clinical characteristics of pSS patients, the results are listed in Table 2. As the results showed, the levels of anti-SSA, anti-SSB, ESR, and *β*_2_-macroglobulin were higher in pSS patients compared with healthy controls. Moreover, the correlation analysis results showed that lnc-DC is positively correlative with anti-SSA (Figure 2(a)), anti-SSB (Figure 2(b)), ESR (Figure 2(c)), and *β*_2_-macroglobulin (Figure 2(d)) expression but not related to the levels of RF, CRP, C3, and C4.

### 3.3. Identification of lnc-DC in Plasma as a Novel Biomarker for pSS

**Figure 3:**
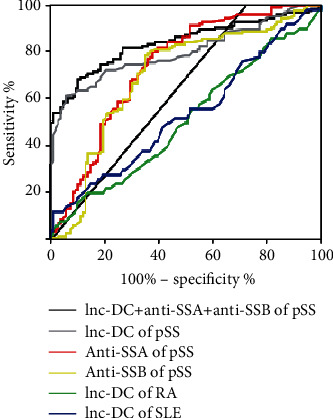
lnc-DC alone or combined with anti-SSA and anti-SSB for the discriminative ability of pSS patients from HC and other autoimmune disease.

In the process of diagnosing pSS plasma biomarkers, the receiver operating characteristic (ROC) curve is used as a performance evaluation method for lnc-DC diagnosis. As shown in the data, an area under the curve (AUC) value for lnc-DC in pSS patients was 0.80. Notably, for lnc-DC combined with anti-SSA and anti-SSB, the AUC value was 0.84. Moreover, at the optimal cutoff value 1.06, the diagnostic sensitivity and specificity were 0.75 and 0.85, respectively. Meanwhile, we also generated ROC curves to analyze the diagnostic value of lnc-DC in SLE and RA to further confirm its specificity. And the AUC values were 0.54 and 0.51 for SLE and RA, respectively (Figure 3 and Table 3). All these results demonstrate that plasma lnc-DC can be used as a novel biomarker for the diagnosis of pSS in clinical.

### 3.4. The lnc-DC Levels in pSS Patients Decreased after Treatment

**Figure 4:**
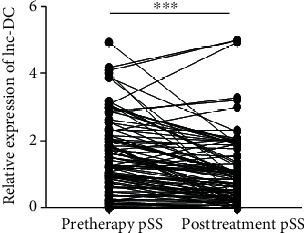
The expression of lnc-DC in plasma of 102 pSS patients before and after treatment. Compared with before treatment, 89 patients showed decreased expression of lnc-DC. ^∗∗∗^*P* < 0.001, by paired sample *t*-test.

Dynamic changes reflecting the patients' condition could provide the clinical guidance for doctors. We therefore tentatively explored the efficacy monitoring capability of lnc-DC in patients receiving drug treatment. The level of plasma lnc-DC in 89 pSS patients, whose treatment with drugs after 6 months, decreased dramatically (Figure 4). The results indicated that dynamic changes of plasma lnc-DC in pSS patients can monitor the treatment efficacy.

## 4. Discussion

Primary Sjögren's syndrome (pSS) is an autoimmune disease with chronic organ-specific characteristics and is characterized by the production of auto-antibodies and the dysfunction of exocrine glands primarily including the lachrymal and salivary glands, which could lead to dry eyes and dry mouth [29, 30]. The incidence of pSS is occult, and the clinical situation varies greatly. Immune thrombocytopenia (ITP) is an immune-mediated disease. If the patient suffers from this disease, there will be abnormalities of platelets, or the production will be damaged, or the damage will be severe, resulting in varying degree of bleeding risk [9]. Although the current research on pSS patients has been explored for many years, the actual research also has many limitations because the lack of cognition of the specific clinical, prevalence, and immunological characteristics of ITP pSS patients during the initial study led to research and has a series of limitations. Usually, patients only start treatment when the condition is very obvious, which leads to the best treatment opportunity may be missed. In order to ensure a good treatment effect, diagnosis and treatment should be timely, and researchers should actively explore new biomarkers.

More and more evidence shows that noncoding RNA, especially microRNAs, plays an important role in the regulation of inflammatory signaling pathways [7, 8]. In recent years, lncRNAs, as a new regulator, are known by reports [31, 32]. Moreover, lncRNAs are widely involved in the gene expression and participate in immune disease [33, 34], and the miRNA-mRNA relationship needs further research, such as the mechanism of miRNA regulation in pSS progression, which might be affected by lnc-DC. However, the specific value application of lncRNAs in pSS is unclear, but the higher level of lnc-DC may participate in pSS without or with ITP.

The results of Wang et al. showed that through lnc-DC, the targets of signal transducer regulation and transcription activator 3 (STAT3) expression can be achieved, thereby modulating the differentiation of dendritic cell [19]. lnc-DC was also reported to mediate the STAT3 expression to regulate differentiation of Th17 cells and functions of T helper cells and B cells [35, 36]. Li et al. found that compared with healthy control, the level of lnc-DC was significantly lower in SLE patients [37]. Our research shows for the first time that the lnc-DC expression is significantly higher than that of plasma pSS patients. Compared with healthy controls, patients have other autoimmune diseases such as systemic lupus erythematosus and rheumatoid arthritis. In addition, we found that lnc-DC plasma levels positively correlated with clinical manifestations, such as increasing the expression of anti-SSA, anti-SSB, ESR, and *β*_2_-microglobulin. Recent studies have demonstrated that lncRNAs may act as effective and noninvasive biomarkers in gynecological diseases [38] and renal diseases [39]. However, it remains to be studied in pSS diagnosis of performance lncRNAs. Our research shows that the plasma lnc-DC has the potential to be the pSS-specific signature lncRNA and could function as a candidate biomarker for pSS. Meanwhile, the AUC of lnc-DC combined with anti-SSA and anti-SSB is much higher than other groups. And the risk score based on lnc-DC could discriminate pSS patients from SLE and RA. This diagnostic efficiency is relatively high that may be caused by the increased expression of DC leads to the specificity lnc-DC high, or lnc-DC regulates the STAT3 expression to mediate Th17 cells, and then, the increased Th17 cells can secrete a variety of cytokines, which leads to the occurrence and development of pSS patients.

However, several limitations in our study must be considered. First, our study is limited to the patients admitted in only one hospital in China, which may restrict the generalizability of the results. Second, the sample size was relatively small. Third, the causal relationship between lncRNAs and pSS revealed by our research is challenging. Thus, the role of lncRNAs in the pSS pathogenesis and development awaits further exploration, both in vivo and in vitro.

In conclusion, our results first demonstrated that plasma lnc-DC level could function as a novel biomarker specifically identifying pSS patients, which is of great importance in the diagnosis of pSS.

## Figures and Tables

**Table 1:** The basic characteristics, clinical manifestations and laboratory findings of pSS patients with and without ITP.

Characteristic	pSS without ITP (*n* = 127)	pSS with ITP (*n* = 22)	t/*χ*^2^/Z	*P* value
Basic characteristics				
Age (years)	48.59 ± 12.98	49.34 ± 15.72	-0.242 ^a^	0.809
Gender (female/male)	121/6	20/2	0.107^b^	0.744
Clinical manifestations				
Dry mouth (%)	85/118 (72.03)	11/20 (55.00)	2.344^b^	0.126
Dry eye (%)	60/118 (50.85)	6/20 (30.00)	2.979^b^	0.084
Arthritis (%)	41/110 (37.27)	3/19 (15.79)	3.327^b^	0.068
Interstitial lung disease (%)	58/108 (53.70)	5/17 (29.41)	3.467^b^	0.063
ESSDAI	4.00 (2.00–6.00)	5.00 (3.00–7.00)	-2.135^c^	0.033
Laboratory findings				
lnc-DC, IU/mL	1.57 ± 0.09	3.56 ± 0.28	-33.041^a^	<0.001
Platelet, ×109/L	234.00 ± 186.00	37.00 ± 33.00	22.384^c^	<0.001
Hemoglobin, g/L	124.00 ± 102.00	117.00 ± 110.00	0.522^c^	0.602
Leukocyte, ×109/L	5.77 ± 3.83	6.05 ± 4.01	-0.776^c^	0.438
Creatinine, mg/dL	46.20 ± 37.20	42.50 ± 29.80	1.985^c^	0.047
ALT, U/L	19.00 ± 16.00	22.00 ± 15.00	-0.950^c^	0.342
AST, U/L	19 ± 13.00	24.00 ± 15.00	-1.232^c^	0.218
Positive ANA (%)	93/116 (80.17)	15/20 (75.00)	0.052^b^	0.819
Positive AHA (%)	2/121 (16.67)	2/18 (11.11)	—	0.081^d^

Data are presented as mean ± SD (standard deviation) or median with 25–75th percentiles, positive number/tested number (%). ESSDAI: European League Against Rheumatism Sjögren's Syndrome Disease Activity Index; ALT: alanine aminotransferase; AST: aspartate aminotransferase; Anna: antinuclear antibody; aha: anti-histone antibody. *P* < 0.05 was considered statistically significant. ^a^Independent sample *t*-test; ^b^chi-squared test; ^c^Mann–Whitney *U* test; ^d^Fisher exact probability method.

**Table 2:** The results of clinical characteristics in HC, pSS, SLE, and RA patients.

Characteristic	HC	pSS patients	SLE patients	RA patients	*F*	*P*
Age	44.27 ± 8.53	48.59 ± 12.98^∗^	47.25 ± 10.33	50.22 ± 14.75^∗^	4.028	0.008
Ro (anti-SSA)	8.22 ± 0.66	13.82 ± 0.56^∗^	7.01 ± 0.77^∗^#	8.12 ± 0.98#&	1915.468	<0.01
La (anti-SSB)	6.78 ± 0.54	10.52 ± 0.44^∗^	7.92 ± 1.33^∗^#	6.39 ± 1.03^∗^#&	607.159	<0.01
ESR (mm/hr)	7.75 ± 0.75	23.46 ± 2.43^∗^	26.33 ± 6.17^∗^#	45.34 ± 14.37^∗^#&	437.691	<0.01
RF (IU/mL)	22.39 ± 14.77	140.29 ± 74.88^∗^	135.45 ± 93.09^∗^	196.77 ± 143.92^∗^#&	69.471	<0.01
Immunoglobulin G (g/L)	9.51 ± 4.33	16.32 ± 8.32^∗^	22.55 ± 9.26^∗^#	13.85 ± 6.90^∗^#&	40.896	<0.001
Immunoglobulin A (g/L)	2.57 ± 1.94	3.42 ± 1.28^∗^	2.33 ± 1.73#	2.42 ± 1.57#	9.111	<0.001
Immunoglobulin E (IU/mL)	69.44 ± 52.15	76.32 ± 58.32	77.89 ± 56.37	70.42 ± 54.61	0.459	0.711
Immunoglobulin M (g/L)	1.47 ± 0.92	1.55 ± 0.93	3.92 ± 2.09^∗^#	4.13 ± 2.41^∗^#	70.399	<0.01
CRP (mg/L)	1.55 ± 0.96	4.02 ± 2.98^∗^	4.55 ± 3.92^∗^	3.19 ± 2.81^∗^&	22.198	<0.01
Complement 3 (g/L)	0.79 ± 0.44	0.92 ± 0.62	1.15 ± 0.62^∗^#	0.96 ± 0.53	4.956	0.002
Complement 4 (g/L)	0.20 ± 0.13	0.28 ± 0.17^∗^	0.32 ± 0.19^∗^	0.25 ± 0.14&	8.392	<0.001
*β*_2_-microglobulin (mg/L)	1.12 ± 0.64	2.81 ± 1.09^∗^	2.54 ± 1.08^∗^	2.01 ± 1.37^∗^#&	57.675	<0.01

ESR: erythrocyte sedimentation rate; RF: rheumatoid factor; CRP: C-reactive protein; *P* < 0.05 was considered statistically significant. One-way ANOVA was used for intergroup comparison, and lSD-T test was used for pairwise comparison as a whole.^∗^ vs. healthy controls *P* < 0.05 # vs. pSS patients *P* < 0.05 & vs. SLE patients *P* < 0.05.

**Table 3:** lnc-DC alone or lnc-DC combined with anti-SSA and anti-SSB for the discriminative ability of pSS patients from HC and other autoimmune disease such as SLE and RA.

Characteristic	AUC	SE	*P* value	95% CI	Sensitivity	Specificity
lnc-DC+antu-SSA+anti-SSB of pSS	0.84	0.03	<0.001	0.79~0.89	78.50	89.91
lnc-DC of pSS	0.80	0.03	<0.001	0.75~0.86	75.42	84.50
Anti-SSA of pSS	0.74	0.05	<0.001	0.68~0.81	72.31	62.39
Anti-SSB of pSS	0.70	0.05	<0.01	0.63~0.77	71.53	64.22
lnc-DC of RA	0.54	0.06	0.464	0.44~0.64	50.00	56.88
lnc-DC of SLE	0.51	0.06	0.836	0.41~0.61	64.09	40.37

## Data Availability

In this study, all the research data involved are presented in this article. All data generated or analyzed are included in this article.
